# Impact of phosphomimetic and non-phosphorylatable mutations of phospholemman on L-type calcium channels gating in HEK 293T cells

**DOI:** 10.1111/jcmm.12484

**Published:** 2015-02-05

**Authors:** Kai Guo, Yue-Peng Wang, Zhi-Wen Zhou, Yi-Bo Jiang, Wei Li, Xiao-Meng Chen, Yi-Gang Li

**Affiliations:** Department of Cardiology, Xin Hua Hospital Affiliated to Shanghai Jiao Tong University School of MedicineShanghai, China

**Keywords:** phospholemman, phosphorylation sites mutation, L-type calcium channels, activation, voltage-dependent inactivation, deactivation

## Abstract

**Background:**

Phospholemman (PLM) is an important phosphorylation substrate for protein kinases A and C in the heart. Until now, the association between PLM phosphorylation status and L-type calcium channels (LTCCs) gating has not been fully understood. We investigated the kinetics of LTCCs in HEK 293T cells expressing phosphomimetic or nonphosphorylatable PLM mutants.

**Methods:**

The LTCCs gating was measured in HEK 293T cells transfected with LTCC and wild-type (WT) PLM, phosphomimetic or nonphosphorylatable PLM mutants: 6263AA, 6869AA, AAAA, 6263DD, 6869DD or DDDD.

**Results:**

WT PLM significantly slowed LTCCs activation and deactivation while enhanced voltage-dependent inactivation (VDI). PLM mutants 6869DD and DDDD significantly increased the peak of the currents. 6263DD accelerated channel activation, while 6263AA slowed it more than WT PLM. 6869DD significantly enhanced PLM-induced increase of VDI. AAAA slowed the channel activation more than 6263AA, and DDDD accelerated the channel VDI more than 6869DD.

**Conclusions:**

Our results demonstrate that phosphomimetic PLM could stimulate LTCCs and alter their dynamics, while PLM nonphosphorylatable mutant produced the opposite effects.

## Introduction

Phospholemman (PLM), a member of the FXYD gene family of small ion transport regulators 1, is abundantly expressed in the cardiac sarcolemma and can modulate Na^+^-K^+^-ATPase (NKA) 2–4 and Na^+^/Ca^2+^ exchanger (NCX) 5–7. Previous studies demonstrated that PLM coimmunoprecipitated LTCCs (Ca_V_1.2 channels) 8 and modulated the important gating process of Ca_V_1.2 channels 8,9. PLM is also a major sarcolemmal substrate for protein kinases A (PKA) and C (PKC) in the myocardium 10,11. When phosphorylated at serine^68^, PLM can stimulate NKA 2,3,12 and inhibit NCX 5,7 in cardiac myocytes. As the activity status of LTCCs is related to the risk of various malignant arrhythmias, such as timothy syndrome (TS) 13–16, modulating PLM phosphorylation might be a potential strategy for preventing and treating arrhythmias. The role of PLM phosphorylation in the regulation of Ca_V_1.2 gating kinetics remains to be elucidated. In this study, we examined the impact of PLM phosphorylation status on Ca_V_1.2 gating kinetics in HEK 293T cells using site-directed mutagenesis and whole-cell patch-clamp electrophysiology techniques.

## Materials and methods

### Construction of WT and mutant PLM

The coding sequence of human PLM was amplified by polymerase chain reaction (PCR) with a His tag on C-terminus, digested with NheI and NotI, and inserted into pCDNA3.1(+)-IRES-GFP vector using the same restriction sites. PLM mutants, 6263AA, 6869AA, AAAA, 6263DD, 6869DD and DDDD, were constructed by PCR-based site-directed mutagenesis (Table S1 shows primers used to introduce the desired mutations into PLM at positions corresponding to amino acids 62, 63, 68 and 69). WT PLM and mutants were confirmed by qualitative restriction map analysis, DNA sequence analysis and Western blot analysis.

### Cell culture and transfection

HEK 293T cells from ATCC (Manassas, VA, USA) were cultured at 37°C and 5%CO_2_ in DMEM-F12 medium (Gibco, CA, USA) supplemented with 10% foetal bovine serum and 1% penicillin-streptomycin. HEK 293T cells were transiently transfected with Hilymax following the manufacturer's instructions (Dojindo Laboratories, Kumamoto, Japan). cDNAs encoding Ca_V_1.2 (α_1_C subunit of LTCCs) 17, the auxiliary subunits, α_2_δ 18 and β_1_b 19 (all subcloned into pCDNA3.1) were cotransfected with either empty vector (EV), WT PLM or mutant PLMs at equal molar ratios.

### Electrophysiology

Whole-cell patch-clamp recordings were performed as described previously 8,9. Briefly, whole-cell currents were recorded at room temperature within 24–48 hrs post-transfection. Pipettes were pulled from borosilicate glass (1B150F-3, World Precision Instruments, Sarasota, FL, USA) using a Narishige PC-10 micropipette puller (Narishige, Japan). The pipette resistance ranged from 3.0 to 4.0 MΩ when the pipette was filled with the internal solution. Ionic currents were recorded in a bath solution containing 130 mM *N*-methyl-d-glucamine (NMG)-aspartate, 10 mM HEPES, 10 mM 4-aminopyridine, 10 mM glucose, 1 mM MgCl_2_ and 10 mM BaCl_2_. The internal solution contained 140 mM NMG-MeSO_3_, 10 mM EGTA, 1 mM MgCl_2_, 4 mM Mg-ATP and 10 mM HEPES. The osmolarity was adjusted to 300 mmol/kg with dextrose, and the pH was adjusted to 7.35. The use of Na^+^, K^+^ and Ca^2+^ free solutions enabled the recording of isolated Ca_V_1.2 currents by eliminating possible contamination from currents originating from the NKA and NCX. The data were acquired using a HEKA EPC10 amplifier and PULSE/PULSEFIT software (ALA Scientific Instruments, Farmingdale, NY, USA). Leak and capacitive transients were corrected by P/4 leak subtraction. The series resistance was typically <8 MΩ and compensated at 70%. The tail currents were sampled at 50 kHz and filtered at 5.0 kHz. All other currents were sampled at 20 kHz and filtered at 3.0 kHz.

### Data analysis

The data were analysed using Fitmaster (ALA Scientific Instruments) and Origin (Originlab, Northampton, MA, USA) software. A one-way anova was used to evaluate the statistical significance. All data were presented as means ± standard errors, and the level of statistical significance was set at *P* < 0.05. Error bars smaller than the symbols do not appear in the figures. The data that significantly differed from WT PLM are indicted with asterisks.

## Results

To test the potential impact of PLM phosphorylation on modulating Ca_V_1.2 channels, the two adjacent phosphorylation sites (S62S63 and S68T69) were replaced with alanine (A) or aspartic acid (D) simultaneously to generate PLM mutants 6263AA, 6869AA, 6263DD and 6869DD. The four potential phosphorylation sites were also replaced with A or D to generate PLM mutants AAAA or DDDD to assess if AAAA or DDDD mutants would further enhance the effects of AA or DD mutants. Whole-cell recordings results showed that WT human and WT canine PLM 8,9 had similar impacts on the Ca_V_1.2 channels (Fig. S1), and the amino acids substitution at potential phosphorylation sites did not negate the ability of PLM to modulate Ca_V_1.2 channels gating (Figs S2–S6, Table S2).

### Aspartic acid substitutions increase the peak of the current density (I_peak_)

**Fig 1:**
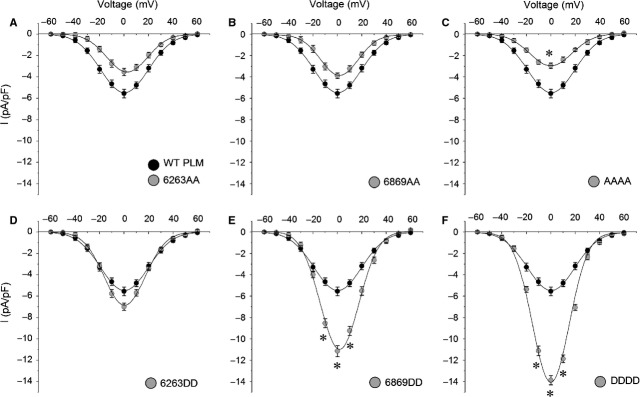
Aspartic acid substitutions within the phosphorylation sites of PLM increase the peak current density. The current-voltage relationship showing the peak current density (I_peak_) was recorded from cells with WT and mutant PLMs (*n* + 6–10). I_peak_ was dramatically increased by PLM phosphomimetic mutants 6263DD, 6869DD, and DDDD and was significantly decreased by the mutant of AAAA, in which the phosphorylation sites were all blocked. * *P *<* *0.05 *versus*WT PLM. Ba^2+^ was used as the charge carrier.

Whole-cell recording results showed that aspartic acid substitution of phosphorylation sites increased I_peak_ (Fig.1D–F), while alanine substitution decreased it (Fig.1A–C). The impact of AAAA and DDDD mutant was more pronounced than that of AA or DD, respectively. Western blot results demonstrated that the expression levels of WT, mutant PLMs and Ca_V_1.2 channels were similar among various mutant groups (Fig. S7), thus, I_peak_ changes might not be induced by changes on the expression levels of WT, mutant PLMs and Ca_V_1.2 channels.

### Phosphorylation sites mutation mediates the effects of PLM on Ca_V_1.2 channels gating

**Fig 2:**
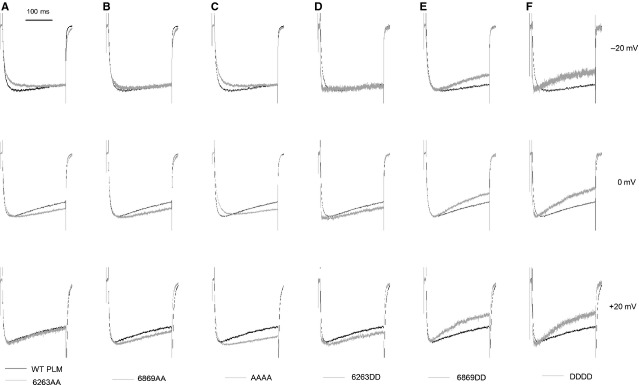
Amino acid substitutions at the PLM phosphorylation sites alter the Ca_V_1.2 channel gating kinetics. (A–F) Whole-cell Ca_V_1.2 currents recorded with WT PLM (black line) or mutant PLMs (grey line) were evoked by 300-msec. depolarizing steps from a holding potential of −90 mV to the voltages indicated on the right. The currents from 6 to 10 cells were normalized and averaged. Ba^2+^ was used as the charge carrier.

To investigate whether PLM phosphorylation status is a crucial determinant of Ca_V_1.2 gating, the normalized currents were superimposed and compared. As shown in Figure2, among the four double mutations, 6263AA most noticeably slowed the channel activation (Fig.2A) and 6869DD most noticeably accelerated the channel VDI (Fig.2E). AAAA further enhanced the effects of 6263AA on the activation slowdown (Fig.2C), and DDDD accelerated VDI more than 6869DD (Fig.2F). Compared with WT PLM, 6869AA and 6263DD did not change the normalized currents visually (Fig.2B and D), but the quantitative analysis showed that they did affect the gating properties of Ca_V_1.2 channels (see below). Thus, the PLM-induced Ca_V_1.2 channels gating changes might be mediated by the amino acid substitutions at the phosphorylation sites.

### Phosphorylation sites mutation at S62S63 affects PLM-induced slowing of Ca_V_1.2 activation

**Fig 3:**
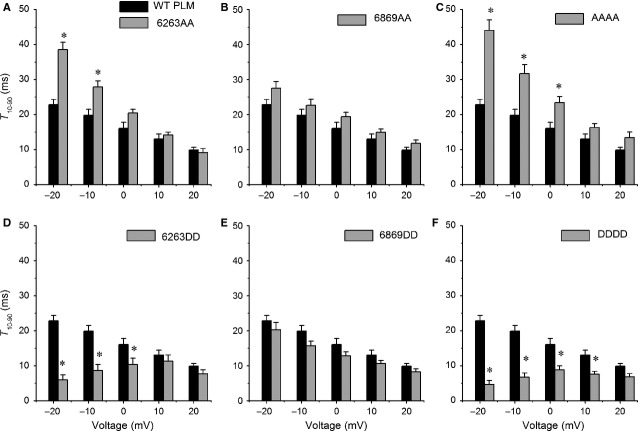
S62S63 phosphorylation affects the PLM-induced Ca_V_1.2 activation slowdown. T_10-90_, the time required to activate from 10% to 90% of the peak currents, was measured from the currents elicited during 300-msec. steps ranging from −20 to +20 mV (10-mV increments, representative currents are depicted in Fig.2). (A) T_10-90_ for 6263AA (grey, *n* + 7) was significantly larger than WT PLM (dark, *n* + 10) at −20 and −10 mV, *P* < 0.05. (B) T_10-90_ for 6869AA (grey, *n* + 6) was larger than WT PLM without significant differences at any observed voltages, *P* > 0.05. (C) T_10-90_ for AAAA (grey, *n* + 7) was significantly larger than WT PLM at −20, −10 and 0 mV, *P* < 0.05. (D) T_10-90_ for 6263DD (grey, *n* + 8) was significantly smaller than WT PLM at −20 and 0 mV, *P* < 0.05. (E) T_10-90_ for 6869DD (grey, *n* + 6) was smaller than WT PLM, without any significant differences at the observed voltages, *P* > 0.05. (F) T_10-90_ for DDDD (grey, *n* + 7) was significantly smaller than WT PLM at −20 to +10 mV, *P* < 0.05. *P < 0.05 *versus*WT PLM.

The activation speed was quantified by measuring the time required for the current to increase from 10% to 90% of the peak current (T_10-90_) 9. T_10-90_ was plotted against step voltages to compare differences between PLM mutants and WT PLM. Compared with those of WT PLM, T_10-90_ values for 6263AA (Fig.3A) and AAAA (Fig.3C) increased significantly at hyperpolarized voltages (close to the channel activation threshold), while T_10-90_ values for 6263DD and DDDD decreased significantly at potentials of −20 mV to 0 mV (Fig.3D and F). T_10-90_ values for 6869AA slightly increased (Fig.3B), while T_10-90_ values for 6869DD slightly decreased (Fig.3E) at all observed potentials.

### Phosphorylation sites mutation at S68T69 affects PLM-induced increase in VDI

**Fig 4:**
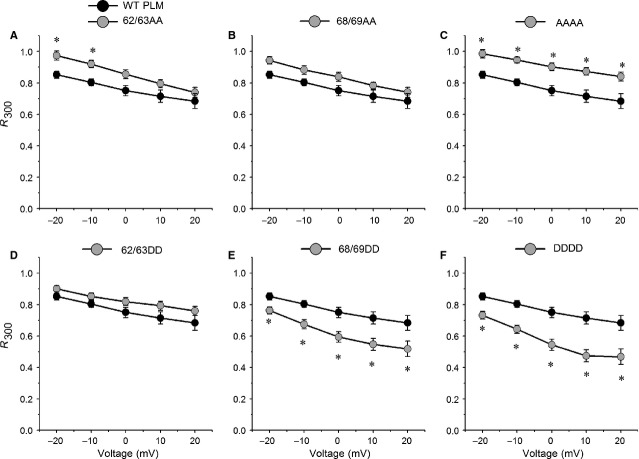
S68T69 phosphorylation affects PLM-induced increases in voltage-dependent inactivation (VDI). R_300_, the fraction of peak current measured at the end of 300-msec. voltage steps to the indicated voltages, was plotted *versus* voltage (representative currents are depicted in Fig.2). (A) R_300_ for 6263AA (*n* + 7) was significantly larger than WT PLM (*n* + 10) at −20 and −10 mV, *P* < 0.05. (B and D) Both of R_300_ for 6869AA (*n* + 6) and 6263DD (*n* + 8) was slightly larger than WT PLM at all observed potentials, *P* > 0.05. (C) R_300_ for AAAA (*n* + 7) was significantly larger than WT PLM at all observed potentials, *P* < 0.05. (E and F) Both of R_300_ for 6869DD (*n* + 6) and DDDD (*n* + 7) was substantially smaller than WT PLM at all observed potentials, *P* < 0.05. *P < 0.05 *versus*WT PLM.

To investigate the inactivation gating, we measured the fraction of the current remaining at the end of 300-ms steps (R_300_) ranging from −20 to +20 mV 9. R_300_ plot *versus* voltages from Ca_V_1.2 currents in the presence of 6869DD showed that this mutant significantly enhanced the ability of PLM to increase VDI, and R_300_ values for 6869DD were significantly smaller than those for WT PLM at all observed voltages (Fig.4E). As expected, DDDD further accelerated PLM-induced increase in VDI, and R_300_ values for DDDD were smaller than those for 6869DD (Fig.4F). R_300_ values for 6869AA were greater than those for WT PLM (Fig.4B). Compared with WT PLM, 6263AA slowed VDI (Fig.4A). AAAA abrogated the PLM-induced increase in VDI, and R_300_ values for AAAA were significantly greater than those for WT PLM at all observed voltages (Fig.4C). The plot of R_300_ for 6263DD was higher than that for WT PLM, and R_300_ values for 6263DD were slightly larger than those for WT PLM (Fig.4D).

### Only AAAA mutant enhanced PLM-induced slowing of deactivation

**Fig 5:**
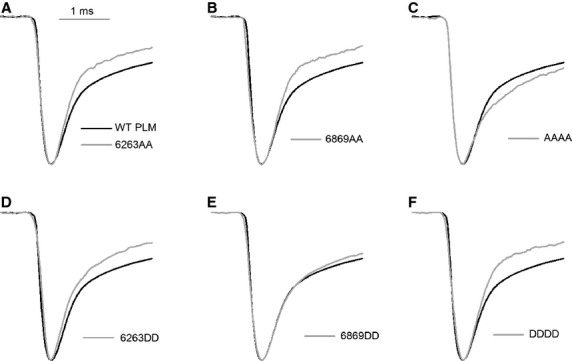
The mutant of AAAA enhances the PLM-induced slowed deactivation. Sample traces for tail currents with WT PLM (dark line) and mutant PLMs (grey line). (A–F) Currents were evoked by repolarizing steps to −50 mV following 100-msec. voltage steps to +80 mV. Tail currents from 6 to 10 cells were normalized and averaged. Ba^2+^ was used as the charge carrier.

**Fig 6:**
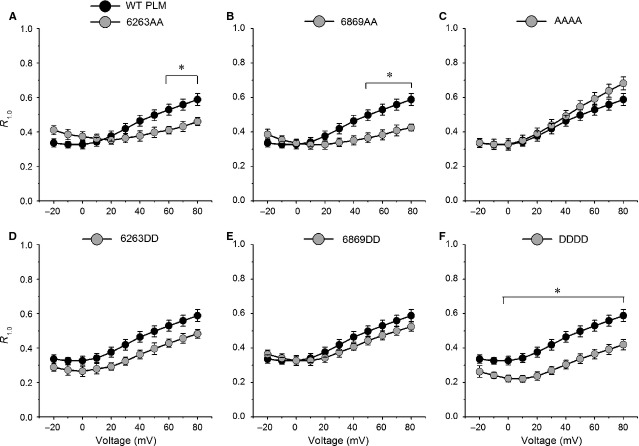
The mutant of DDDD speeds Ca_V_1.2 channel deactivation. R_1.0_, the fraction of current remaining 1 msec. after the peak tail current for WT PLM and mutant PLM, was plotted against step voltages (representative currents are depicted in Fig.5). (A) R_1.0_ for 6263AA (*n* + 7) was smaller than WT PLM (*n* + 10) at +60 to +80 mV, *P* < 0.05. (B) R_1.0_ for 6869AA (*n* + 6) was smaller than WT PLM at +50 to +80 mV, *P* < 0.05. (C) R_1.0_ for AAAA (*n* + 7) was slightly larger than WT PLM, *P* > 0.05. (D) R_1.0_ for 6263DD (*n* + 8) was smaller than WT PLM at all observed voltages, but there were no significant differences, *P* > 0.05. (E) R_1.0_ for 6869DD (*n* + 6) was comparable to WT PLM, *P* > 0.05. (F) R_1.0_ for DDDD (*n* + 7) was smaller than WT PLM at 0 to +80 mV, *P* < 0.05. *P < 0.05 *versus*WT PLM.

Tail currents were evoked by repolarizing to −50 mV following 100-ms depolarizing steps ranging from −20 to +80 mV. The currents at +80 mV were normalized and superimposed to highlight the effects of PLM mutants comparing the effects of WT PLM on the time course of deactivation. All mutant PLMs except AAAA mutant suppressed PLM-induced deactivation slowdown (Fig.5A–F). These effects were quantified by measuring the relative tail current amplitude at 1 msec. after the peak tail current (R_1.0_) 9. These isochronic measurements of deactivation were illustrated by R_1.0_ plots *versus* step voltages (Fig.6). R_1.0_ values for AAAA tended to be larger than those for WT PLM (*P* > 0.05, Fig.6C). R_1.0_ values for DDDD were significantly smaller than those for WT PLM at voltages ranging from 0 to +80 mV (Fig.6F). R_1.0_ values for 6869AA were significantly smaller than those for WT PLM at voltages ranging from +50 to +80 mV (Fig.6B). R_1.0_ values for 6263AA were significantly smaller than those for WT PLM at voltages ranging from +60 to +80 mV (Fig.6A). R_1.0_-voltage relationship for 6869DD was similar as that of WT PLM (Fig.6E). R_1.0_ values for 6263DD tended to be smaller compared with those for WT PLM (*P* > 0.05, Fig.6D).

## Discussion

The major findings of this study are: (*i*) PLM phosphomimetic mutants 6263DD, 6869DD and DDDD increased while nonphosphorylatable PLM mutants 6263AA, 6869AA, AAAA decreased the peak of Ca_V_1.2 current density; (*ii*) 6263DD suppressed while 6263AA enhanced PLM-induced activation slowdown; (*iii*) 6869DD enhanced PLM-induced increased VDI, while 6869AA had the same effect on VDI as WT PLM; and (*iv*) only the AAAA mutant enhanced PLM-induced slowing of channel deactivation. Thus, the phosphorylation sites in PLM are important for fine-tuning the gating kinetics of Ca_V_1.2 channels and could be involved in the kinase-dependent regulation of these channels.

### PLM phosphomimetic mutants stimulated LTCCs

Aspartic acid was used to substitute these potential phosphorylation sites in human PLM, as commonly performed 20–23. Compared with WT PLM, all of phosphomimetic PLM mutants, 6263DD, 6869DD and DDDD, increased the current amplitude, although Western blot analysis indicated that the expression level of Ca_V_1.2 channels remained unchanged (Fig. S7). PLM is a single transmembrane-spanning protein that inserts into the sarcolemma. Based on nuclear magnetic resonance spectroscopic studies, the purified PLM consists of four α-helices, and the forth helix, H4 (residues 60–68) in the C-terminus, is connected to the third helix, H3 (residues 39–45), by a flexible linker 24,25. The forth helix is supposed to orientate with the negatively charged phospholipids of the membrane 24. We speculate that when PLM is phosphorylated at the phosphorylation sites, the orientation of the cytoplasmic tail might shift and then unlock the connection of PLM and Ca_V_1.2 channels. This speculation is supported by the fact that Ca_V_1.2 activation was significantly increased by 6263DD and DDDD (Fig.3D and F). To confirm the mechanism, additional experiments are warranted, such as Glutathione S-transferase pull-down experiments to examine the exact connection sites between PLM and Ca_V_1.2 channel.

### The phosphomimetic mutation at S62S63 and S68T69 mediates PLM-induced Ca_V_1.2 channel activation and inactivation, respectively

We observed that 6263AA slowed the channel activation and inactivation more than WT PLM at voltages of −20 mV and −10 mV (Figs3A and 4A). As channel inactivation could impact the measurement of channel activation kinetics 9,26, it is possible that the enhanced slowing of Ca_V_1.2 channel activation by 6263AA is an indirect consequence of the loss of PLM-induced VDI, indicating that 6263AA might not slow activation more than WT PLM, but it only appears to slow activation because VDI is attenuated. It is supported by the effects of AAAA (as they are the same effects as 6263AA) (Figs3C and 4C), and also supported by the associated enhancement of VDI and speeding of activation by the DDDD mutant (Figs3F and 4F). However, the impact that 6263DD has on VDI is similar to that of WT PLM (Fig.4D), but 6263DD abrogates PLM-induced slowed activation (Fig.3D), suggesting that *phosphomimetic mutation at S62S63* mediates PLM-induced channel activation without affecting inactivation. Accordingly, 6869DD promotes the channel VDI as fast as DDDD (Fig.4E), but the activation kinetics of Ca_V_1.2 currents with 6869DD are similar to those of WT PLM (Fig.3E). Combined with results showing that 6869AA did not alter PLM-induced activation or VDI (Figs3B and 4B), it appears that *phosphomimetic mutation at* S68T69 mediates PLM-induced VDI without affecting activation.

### The phosphomimetic mutations mediate PLM-induced Ca_V_1.2 channel deactivation

Ca_V_1.2 channels have been shown to exhibit modal gating behaviour 27,28, and PLM promotes mode 2 gating [high open probability (*P*_O_)] by enhancing the voltage- and time-dependent slowing of deactivation 8. Of the six mutants investigated in this study, only the AAAA mutant, which has all four potential phosphorylation sites substituted by alanine, slowed deactivation, similar to WT PLM (Fig5C and 6C). The other five mutant PLMs prevented PLM-induced slowed deactivation, although the effects of 6263DD and 6869DD did not significantly differ from that of WT PLM. As slowed deactivation has been predicted to enhance the relative Ca^2+^ entry during the repolarization phase of the cardiac action potential by favouring the high *P*_O_ state (mode 2), which is arrhythmogenic 8,13, we speculate that PLM phosphorylation might help protect the Ca^2+^ overload under certain conditions, such as ischaemia. However, it is unclear why 6869AA reduced the PLM-induced slowing deactivation more than 6869DD, thus, single-channel experiments are necessary to confirm the effects of *phosphomimetic mutations* on Ca_V_1.2 channel mode switching and Ca^2+^ influx.

The changes of Ca_V_1.2 gating properties mediated by phosphomimetic PLM are not the same as the changes mediated by PKA. For example, PKA-mediated up-regulation of Ca_V_1.2 activity is dependent on the increase in channel open probability 29, however, the nonphosphorylatable mutant AAAA favoured the high probability. For another example, PKA decreased the VDI *in situ* 30, but the phosphomimetic PLM 6869DD increased the VDI. Zhang, *et al*. showed that phorbol12-myristate13-acetate (PMA, PKC activators) could increase the magnitude of the I_NCX_ in HEK 293 cells expressing NCX alone, however, this effect was much smaller in HEK 293 cells co-expressing NCX and PLM. Thus, the stimulatory effects of PMA on NCX were attenuated by increased PLM phosphorylation 7. At this point, the direct effect of PKA on LTCC might not be the same as the indirect effect of PKA mediated by PLM and the effect of phosphomimetic PLM on LTCC. Present data were obtained based on the gating properties of Ca_V_1.2 channels regulated by the phosphomimetic and nonphosphorylatable mutations of PLM. As the effects of PLM mutations on LTCCs modulation were studied in the heterogonous expression systems in HEK 293T cells, the determination of the physiological role of PLM phosphorylation (by PKA or not) and its regulation of LTCCs is beyond the scope of this manuscript and future *in vivo* experiments are warranted to explore the issue. Moreover, the effects of phosphomimetic mutants should be compared with the cAMP-PKA effects on LTCC in intact cardiomyocytes.

### Physiological relevance of PLM phosphorylation in Ca_V_1.2 channels gating

It has been shown that changes in Ca_V_1.2 channels gating, such as impaired VDI, profoundly affect cardiac function 13–16. TS is a multi-organ disorder caused by a single mutation, G406R (TS mutation), of an alternatively spliced human Ca_V_1.2 calcium channel containing exon 8a 14. This TS mutation can lead to lethal arrhythmias, which are thought to be caused by impaired VDI 14,15. Our results showed that 6869DD and DDDD could enhance PLM-induced increases in VDI, so we speculate that it is possible that PLM phosphorylation might restore TS-impaired VDI. In fact, LTCC blockers, such as gallopamil, verapamil and diltiazem, can enhance VDI 31,32 and currently serve as clinically effective medications for certain arrhythmias that result from Ca_V_1.2 channel-induced early afterdepolarization 33,34. Therefore, PLM could be a promising drug target for treating arrhythmias caused by disrupted I_Ca(L)_ inactivation. However, the exact role of PLM phosphorylation in arrhythmias due to LTCCs dysfunction remains largely unclear. For example, the nonphosphorylatable mutants, 6263AA and AAAA, decreased channels activation. It seems that these mutants would prevent excessive Ca^2+^ from entering into cardiac myocytes and reduce the occurrence of arrhythmias. But their effects on activation were not evident at 0 to +20 mV, which is near the action potential peak and early plateau. Additionally, both 6263AA and AAAA abrogated PLM-induced increase in VDI, so the amount of intracellular Ca^2+^ was unknown. Therefore, it would be important to explore the impact of PLM phosphorylation status on Ca_V_1.2 channels gating in cells, which have undergone hypoxic or mechanical stress conditions *in vitro* and hypoxia and ischaemia situations, or arrhythmia animal models *in vivo*. Such studies may further enhance our understanding on this issue. The present study, however, presented data to show the impact of *phosphomimetic and nonphosphorylatable PLM mutants* on Ca_V_1.2 channels gating in HEK 293T cells. Future studies are warranted to determine the physiological role of PLM phosphorylation and its regulation of LTCCs in an *in vivo* environment.

In conclusion, our results demonstrate that *phosphomimetic PLM* stimulates LTCCs and alters their dynamics, while PLM nonphosphorylatable mutants lead to opposite effects on LTCCs. Thus, the phosphorylation sites in PLM are important for fine-tuning the gating kinetics of Ca_V_1.2 channels.

## Supporting Information

**Figure S1** Effects of WT human PLM on Ca_V_1.2 channels gating.

**Figure S2** Amino acid substitutions at the PLM phosphorylation sites alter Ca_V_1.2 channel gating kinetics.

**Figure S3** S62S63 phosphorylation affects the PLM-induced Ca_V_1.2 activation slowdown.

**Figure S4** S68T69 phosphorylation affects PLM-induced increases in voltage-dependent inactivation (VDI).

**Figure S5** The mutant of AAAA enhances the PLM-induced slowed deactivation.

**Figure S6** The mutant of DDDD speeds Ca_V_1.2 channel deactivation.

**Figure S7** Amino acid substitutions within the phosphorylation sites of PLM do not alter the Ca_V_1.2 channel or PLM expression levels.

**Data S1** Amino acids substitution at potential phosphorylation sites did not affect the ability of PLM to modulate CaV1.2 channels gating.
