# Supplementary figures and images for: Impact of phosphomimetic and non-phosphorylatable mutations of phospholemman on L-type calcium channels gating in HEK 293T cells
Source: J Cell Mol Med. 2015 Feb 5;19(3):642–50. doi: 10.1111/jcmm.12484 (PMC4369820; doi:10.1111/jcmm.12484)

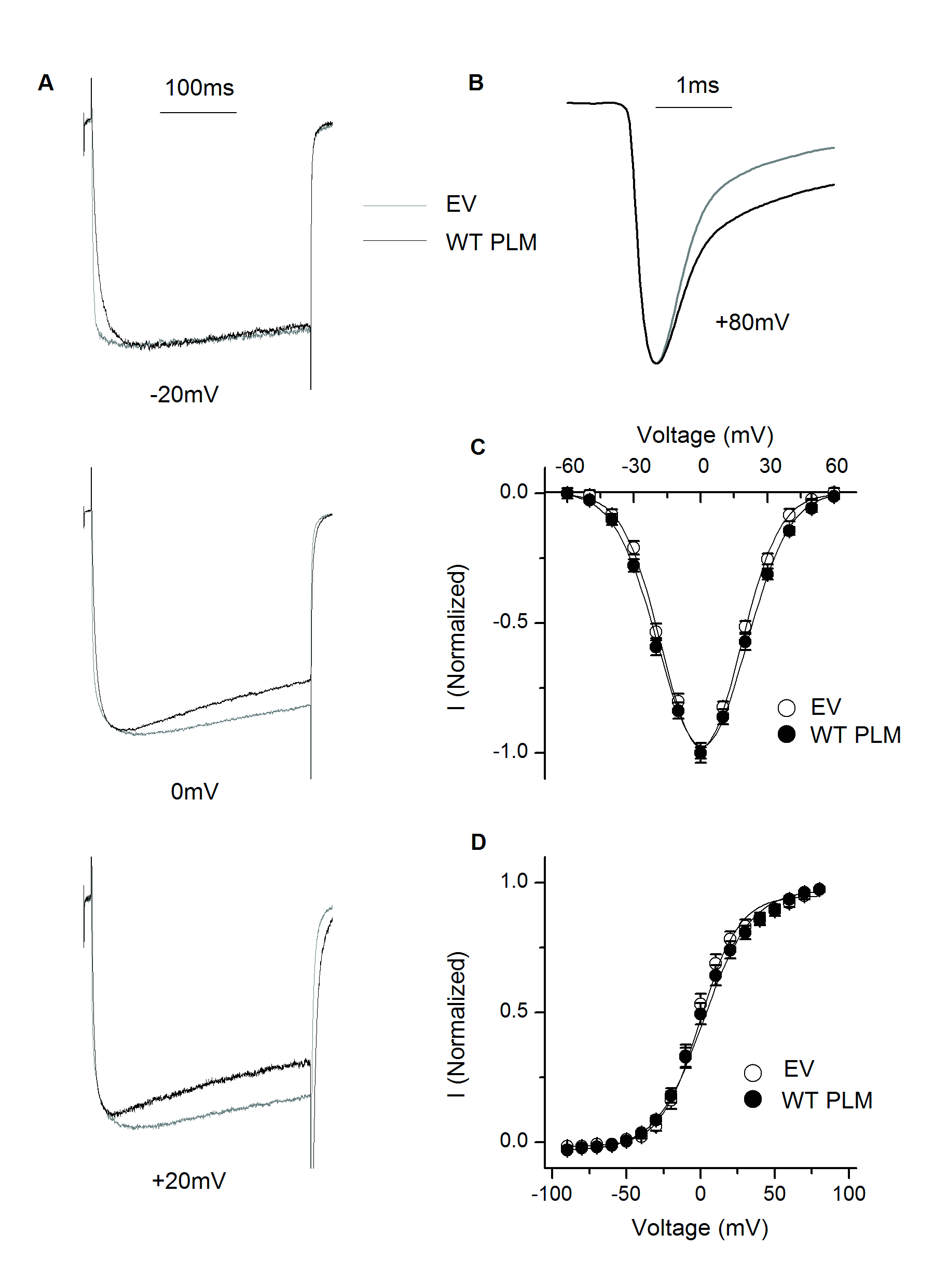

Supplement: Supplementary file 1 [file jcmm0019-0642-sd1.tif]

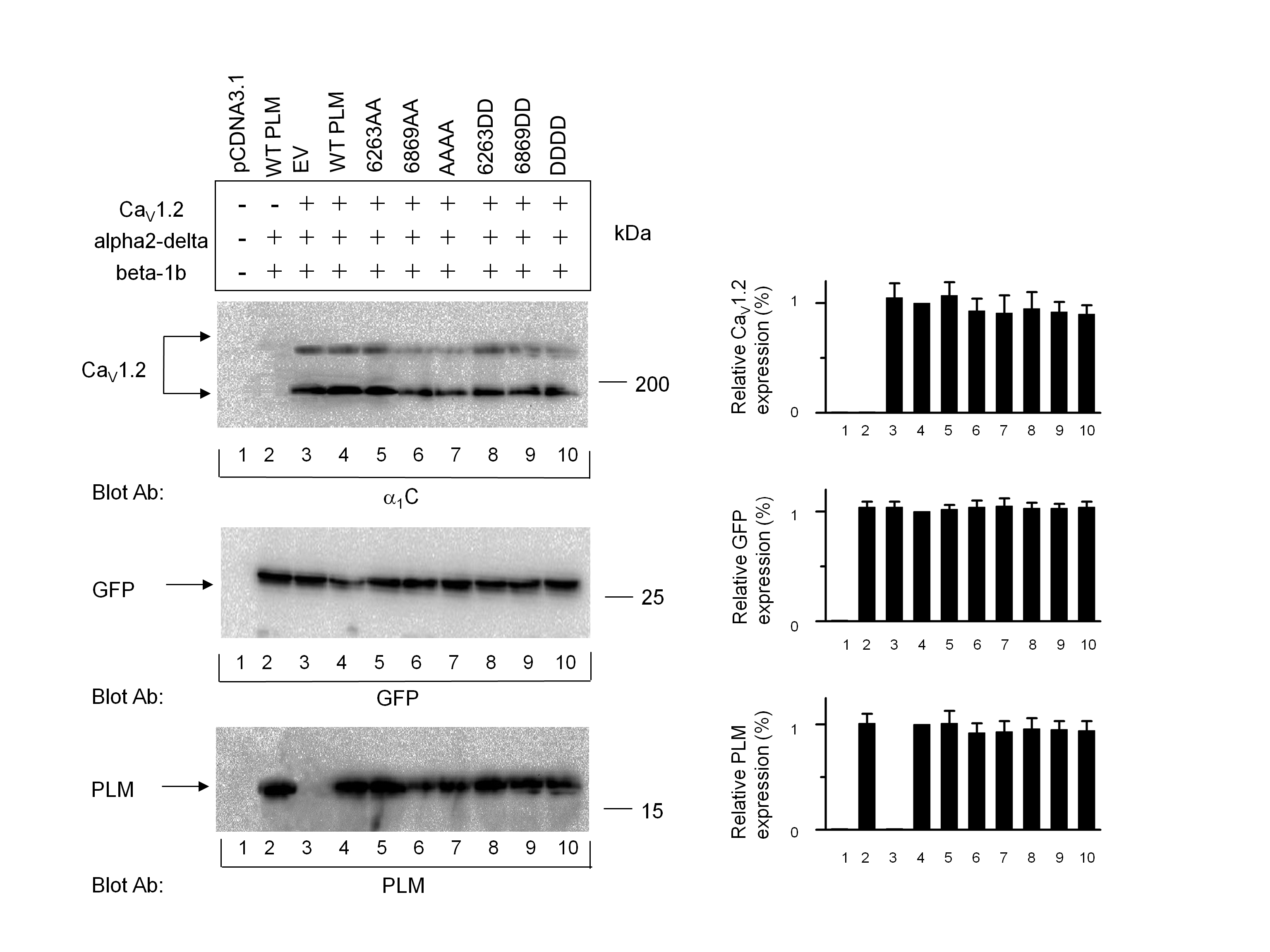

Supplement: Supplementary file 7 [file jcmm0019-0642-sd7.tif]
