# Supplementary material for: Impact of phosphomimetic and non-phosphorylatable mutations of phospholemman on L-type calcium channels gating in HEK 293T cells
Source: J Cell Mol Med. 2015 Feb 5;19(3):642–50. doi: 10.1111/jcmm.12484 (PMC4369820; doi:10.1111/jcmm.12484)
Supplement: Supplementary file 8 [file jcmm0019-0642-sd8.docx]

**Supporting Information**

**Methods**

*Western blot analysis*. Cells were harvested 24h after transfection in lysis buffer[[1](#_ENREF_1)]. Cell lysates were centrifuged at 14,000g at 4ºC for 15min. The protein concentration was measured using the bicinchonic acid (BCA) protein assay reagent (Thermo Fisher Scientific, Waltham, MA), and 30 µg samples of total protein in 1×loading buffer were incubated in boiling water for 5min, and then subjected to polyacrylamide gel electrophoresis, transferred to a polyvinylidene fluoride membrane (PVDF), and blotted with primary (overnight at 4ºC), then secondary (1 hour at room temperature) antibodies. Proteins were detected using ECL regents (Thermo Fisher Scientific, Waltham, MA). Rabbit polyclonal anti-Ca_V_1.2 (Calbiochem, San Diego, CA), rabbit polyclonal anti-PLM (Abcam, Cambridge, MA), rabbit polyclonal anti-GFP (Santa Cruz, Dallas, Texas), and goat anti-rabbit HRP (Cell Signaling Technology, Danvers, MA) were used.

**Results**

Recent reports described that PLM could modulate Ca_V_1.2 channels gating in HEK 293 cells[[1](#_ENREF_1),[2](#_ENREF_2)]. They demonstrated that PLM slowed both activation and deactivation and accelerated voltage-dependent inactivation (VDI) of Ca_V_1.2 channels gating. These results were obtained from the whole-cell recordings (Ba^2+^ as the charge carrier) in the presence or absence of WT canine PLM. Human PLM was used in the present study. Four amino acids were different in human PLM compared with canine PLM (A2, Q4, R17, and I22 for canine PLM and S2, K4, Q17, and V22 for human PLM). As shown in Figure S1, WT human PLM modulated the Ca_V_1.2 channels in the same way as WT canine PLM[[1](#_ENREF_1),[2](#_ENREF_2)]. Whole-cell Ca_V_1.2 currents, recorded with EV and WT PLM, were evoked by 300-ms depolarizing steps from a holding potential of -90 mV to the indicated voltages. The sample traces showed that channel activation with WT PLM was slowed at -20 mV. This effect disappeared with increasing voltages, and the channel VDI was increased at more depolarized voltages (Figure S1A). Figure S1B shows superimposed tail currents from Ca_V_1.2 channels with EV and WT PLM to highlight the effects of PLM on deactivation. The tail currents were evoked by repolarizing steps to -50 mV following 100-ms depolarizing steps ranging from -20 to +80 mV. At all of the measured voltages, WT PLM slowed channel deactivation (Figure S1B shows the results at +80 mV). The current-voltage relationships (Figure S1C) and activation versus voltage relationships (Figure S1D) were identical between WT PLM and EV.

To demonstrate that the mutations did not prevent PLM from modulating Cav1.2 channel gating, the normalized Ca_V_1.2 currents with empty vector (EV), WT PLM, or PLM mutants are shown in figures S2 and S5. Compared with EV (gray, n=9), WT PLM (dark, n=10) slowed Ca_V_1.2 activation at -20 mV, whereas 6263AA (blue, n=7) and AAAA (blue, n=7) slowed it more than WT PLM at that potential (Figure S2A and S2C). There were significant differences when measured with T_10-90_ (Figure S3A and S3C). Compared with EV, WT PLM accelerated voltage-dependent inactivation (VDI), whereas 6869DD (blue, n=6) and DDDD (blue, n=7) enhanced the PLM-induced acceleration of VDI (Figure S2E and S2F). There were significant differences when measured with R_300_ (Figure S4E and S4F). Compared with the other PLM mutants, 6869AA (blue, n=6) and 6263DD (blue, n=8) did not obviously change the current shape (Figure S2B and S2D), but they did modulate Ca_V_1.2 gating. Compared with EV, 6869AA slowed Ca_V_1.2 activation significantly at -20 and -10 mV (Figure S3B) and also slowed deactivation significantly at -20 mV~+10 mV (Figure S6B). Compared with EV, 6263DD accelerated Ca_V_1.2 activation significantly at +10 mV (Figure S3D). Ca_V_1.2 gating parameters in the absence and presence of WT and mutant PLM were listed in Table S2.

The expression levels of WT, mutant PLMs and Ca_V_1.2 channels were assessed by Western blot analysis. As shown in Figure S7, the expression levels of Ca_V_1.2 and PLM did not differ significantly among various mutant groups.

References

1. **Guo K, Wang X, Gao G, *et al*.** Amino acid substitutions in the FXYD motif enhance phospholemman-induced modulation of cardiac L-type calcium channels. *Am J Physiol Cell Physiol*. 2010; 299: C1203-11.

2. **Wang X, Gao G, Guo K, *et al*.** Phospholemman modulates the gating of cardiac L-type calcium channels. *Biophys J*. 2010; 98: 1149-59.

Table S1. Primer sequences

| PLM mutants | Forward primer | Reverse primer |
| --- | --- | --- |
| 6263AA | 5’-GGG AAC TTT CCG CGC TGC CAT CCG CCG TCT GTC CAC CCG C-3’ | 5’- ACA GAC GGC GGA TGG CAG CGC GGA AAG TTC CCT CCT CTT C-3’ |
| 6869AA | 5’- CAT CCG CCG TCT GGC TGC CCG CAG GCG GCA TCA TCA TCA T-3’ | 5’- GAT GCC GCC TGC GGG CAG CCA GAC GGC GGA TGG AGC TGC G-3’ |
| AAAA | 5’- GCT GCC ATC CGC CGT CTG GCT GCC CGC AGG CGG CAT CAT CAT CAT CAT CAT TAG-3’ | 5’- GGC AGC CAG ACG GCG GAT GGC AGC GCG GAA AGT TCC CTC CTC TTC ATC GGG TTC-3’ |
| 6263DD | 5’- GGG AAC TTT CCG CGA TGA CAT CCG CCG TCT GTC CAC CCG C-3’ | 5’- ACA GAC GGC GGA TGT CAT CGC GGA AAG TTC CCT CCT CTT C-3’ |
| 6869DD | 5’- CAT CCG CCG TCT GGA TGA CCG CAG GCG GCA TCA TCA TCA T-3 | 5’- GAT GCC GCC TGC GGT CAT CCA GAC GGC GGA TGG AGC TGC G-3’ |
| DDDD | 5’- GAT GAC ATC CGC CGT CTG GAT GAC CGC AGG CGG CAT CAT CAT CAT CAT CAT TAG-3’ | 5’- GTC ATC CAG ACG GCG GAT GTC ATC GCG GAA AGT TCC CTC CTC TTC ATC GGG TTC-3’ |

Table S2. Ca_V_1.2 gating parameters in the absence and presence of wild-type and mutant PLM

|  | EV | WT PLM | 6263AA | 6869AA | AAAA | 6263DD | 6869DD | DDDD |
| --- | --- | --- | --- | --- | --- | --- | --- | --- |
| T_10-90_(-20mV, ms) | 6.5±0.9^9^ | 22.9±1.4^10^ | 38.6±2.0^7/*^ | 27.5±1.9^6^ | 44.0±3.2^7/*^ | 6.0±1.4^8/*^ | 20.2±2.1^6^ | 4.6±0.5^7/*^ |
| R_1.0_(+80mV) | 0.37±0.03^9^ | 0.57±0.08^10^ | 0.46±0.02^7/*^ | 0.43±0.03^6/*^ | 0.68±0.09^7^ | 0.48±0.05^8^ | 0.52±0.05^6^ | 0.42±0.03^7/*^ |
| R_300_(+20mV) | 0.83±0.07^9^ | 0.68±0.06^10^ | 0.74±0.09^7^ | 0.76±0.08^6^ | 0.84±0.05^7/*^ | 0.76±0.07^8^ | 0.52±0.03^6/*^ | 0.47±0.05^7/*^ |
| I_peak_ (pA/pF) | -5.38±0.12^9^ | -5.55±0.41^10^ | -3.53±0.36^7^ | -3.88±0.27^6^ | -2.95±0.26^7/*^ | -6.99±0.32^8^ | -11.14±0.52^6/*^ | -13.87±0.43^7/*^ |

T_10-90_, time for currents to activate from 10% to 90% of the peak current; R_1.0_, relative tail current amplitude at 1 ms; R_300_, fraction of current remaining at the end of 300-ms steps; I_peak_, the peak of current density; EV, empty vector; WT PLM, wild-type phospholemman; Values that are significantly different from WT PLM are indicated with an asterisk. The sample size for each experiment is indicated in superscript beside the data.

**Figure legends**

**Figure S1. Effects of WT human PLM on Ca_V_1.2 channels gating.** A: Whole-cell Ca_V_1.2 currents recorded with empty vector (EV, n=9, gray line) and wild-type human PLM (WT PLM, n=10, dark line) were evoked by 300-ms depolarizing steps from a holding potential at -90 mV to the indicated voltages. B: Sample traces for tail currents with EV and WT PLM. The currents were evoked using repolarizing steps to -50 mV following 100-ms voltage steps to +80 mV. C: Current-voltage relationships were generated for EV and WT PLM by a series of 300-ms step pulses ranging from -60 to +60 mV from a holding potential of -90 mV. D: Steady-state activation curves for EV and WT PLM were measured at -50 mV after a series of 100-ms step pulses from -90 to +80 mV, and the data were fitted with a Boltzmann equation (smooth lines). Ba^2+^ was used as the charge carrier. The observed effects of WT human PLM on Ca_V_1.2 channels gating were similar as previous reported effects of WT canine PLM [[1](#_ENREF_1),[2](#_ENREF_2)].

**Figure S2. Amino acid substitutions at the PLM phosphorylation sites alter Ca_V_1.2 channel gating kinetics.** A-F: Whole-cell Ca_V_1.2 currents recorded with EV (gray), WT PLM (black), or mutant PLMs (blue) were evoked by 300-ms depolarizing steps from a holding potential of -90 mV to the voltages indicated on the right. The currents from 6-10 cells were normalized and averaged. Ba^2+^ was used as the charge carrier. EV, empty vector.

**Figure S3. S62S63 phosphorylation affects the PLM-induced Ca_V_1.2 activation slowdown.** T_10-90_, the time required to activate from 10% to 90% of the peak currents, was measured from the currents elicited during 300-ms steps ranging from -20 to +20 mV (10-mV increments, representative currents are depicted in Figure S2). A: T_10-90_ for 6263AA (blue, n=7) was significantly larger than EV (gray, n=9) and WT PLM (dark, n=10) at -20 and -10 mV, *P*<0.05. B: T_10-90_ for 6869AA (blue, n=6) was significantly larger than EV at -20 and -10 mV, *P*<0.05. C: T_10-90_ for AAAA (blue, n=7) was significantly larger than EV and WT PLM at -20, -10 and 0 mV, *P*<0.05. D: T_10-90_ for 6263DD (blue, n=8) was significantly smaller than EV at +10 mV and smaller than WT PLM at -20, -10 and 0 mV, *P*<0.05. E: T_10-90_ for 6869DD (blue, n=6) was significantly larger than EV at -20 mV and smaller than EV at +10 mV, *P*<0.05. F: T_10-90_ for DDDD (blue, n=7) was significantly smaller than EV at +10 mV and smaller than WT PLM at -20 to +10 mV, *P*<0.05. * *P*<0.05 versus EV. # *P*<0.05 versus WT PLM. EV, empty vector.

**Figure S4. S68T69 phosphorylation affects PLM-induced increases in voltage-dependent inactivation (VDI).** R_300_, the fraction of peak current measured at the end of 300-ms voltage steps to the indicated voltages, was plotted versus voltage (representative currents are depicted in Figure S2). A: R_300_ for 6263AA (blue, n=7) was significantly larger than WT PLM (black, n=10) at -20 and -10 mV, *P*<0.05. B and D: Neither R_300_ for 6869AA (blue, n=6) nor that for 6263DD (blue, n=8) significantly differed from EV (gray, n=9) or WT PLM at any observed potential, *P*>0.05. C: R_300_ for AAAA (blue, n=7) was significantly larger than WT PLM at all observed potentials, *P*<0.05. E and F: R_300_ for both 6869DD (blue, n=6) and DDDD (blue, n=7) were substantially smaller than EV and WT PLM at all observed potentials, *P*<0.05. * *P*<0.05 versus EV. # *P*<0.05 versus WT PLM. EV, empty vector.

**Figure S5. The mutant of AAAA enhances the PLM-induced slowed deactivation.** Sample traces for tail currents with EV (gray), WT PLM (dark), and mutant PLMs (blue). A-F: Currents were evoked by repolarizing steps to -50 mV following 100-ms voltage steps to +80 mV. Tail currents from 6-10 cells were normalized and averaged. Ba^2+^ was used as the charge carrier. EV, empty vector.

**Figure S6. The mutant of DDDD speeds Ca_V_1.2 channel deactivation.** R_1.0_, the fraction of current remaining 1 ms after the peak tail current, was plotted against step voltages (representative currents are depicted in Figure S5). A: R_1.0_ for 6263AA (blue, n=7) was larger than EV (gray, n=9) at -20 to +10 mV and smaller than WT PLM (black, n=10) at +60 to +80 mV, *P*<0.05. B: R_1.0_ for 6869AA (blue, n=6) was larger than EV at -20 to +10 mV and smaller than WT PLM at +50 to +80 mV, *P*<0.05. C: R_1.0_ for AAAA (blue, n=7) was significantly larger than EV at all observed voltages, *P*<0.05. D: R_1.0_ for 6263DD (blue, n=8) was not significantly different from either EV or WT PLM at any observed voltage, *P*>0.05. E: R_1.0_ for 6869DD (blue, n=6) was significantly larger than EV at all observed voltages, *P*<0.05. F: R_1.0_ for DDDD (blue, n=7) was smaller than WT PLM at 0 to +80 mV, *P*<0.05. * *P*<0.05 versus EV. # *P*<0.05 versus WT PLM. EV, empty vector.

**Figure S7. Amino acid substitutions within the phosphorylation sites of PLM do not alter the Ca_V_1.2 channel or PLM expression levels.** Lysates from HEK 293T cells expressing Ca_V_1.2 channels and EV, WT PLM and mutant PLM were subjected to polyacrylamide gel electrophoresis. The separated proteins were transferred to PVDF membranes and were probed with anti-Ca_V_1.2, anti-PLM and anti-GFP antibodies. Immunoblots were quantified using Bio-Rad Image software (Hercules, CA, USA) and are shown on the right side of the panel. The data were normalized to the conditions depicted in lane 4 (Western blot analysis was repeated 5 times).
